# Seasonal patterns of spatial fidelity and temporal consistency in the distribution and movements of a migratory ungulate

**DOI:** 10.1002/ece3.7650

**Published:** 2021-05-14

**Authors:** Kyle Joly, Eliezer Gurarie, D. Alexander Hansen, Matthew D. Cameron

**Affiliations:** ^1^ Gates of the Arctic National Park and Preserve Arctic Inventory and Monitoring Network National Park Service Fairbanks AK USA; ^2^ Department of Biology University of Maryland College Park MD USA; ^3^ Division of Wildlife Conservation Alaska Department of Fish and Game Kotzebue AK USA

**Keywords:** adhesion, caribou, conservation, fidelity, migration, productivity, *Rangifer*, space use, Western Arctic Herd

## Abstract

How animals use their range can have physiological, ecological, and demographic repercussions, as well as impact management decisions, species conservation, and human society. Fidelity, the predictable return to certain places, can improve fitness if it is associated with high‐quality habitat or helps enable individuals to locate heterogenous patches of higher‐quality habitat within a lower‐quality habitat matrix. Our goal was to quantify patterns of fidelity at different spatial scales to better understand the relative plasticity of habitat use of a vital subsistence species that undergoes long‐distance migrations. We analyzed a decade (2010–2019) of GPS data from 240 adult, female Western Arctic Herd (WAH) caribou (*Rangifer* *tarandus*) from northwest Alaska, U.S.A. We assessed fidelity at 2 spatial scales: to site‐specific locations within seasonal ranges and to regions within the herd's entire range by using 2 different null datasets. We assessed both area and consistency of use during 6 different seasons of the year. We also assessed the temporal consistency of migration and calving events. At the scale of the overall range, we found that caribou fidelity was greatest during the calving and insect relief (early summer) seasons, where the herd tended to maximally aggregate in the smallest area, and lowest in winter when the seasonal range is largest. However, even in seasons with lower fidelity, we found that caribou still showed fidelity to certain regions within the herd's range. Within those seasonal ranges, however, there was little individual site‐specific fidelity from year to year, with the exception of summer periods. Temporally, we found that over 90% of caribou gave birth within 7 days of the day they gave birth the previous year. This revealed fairly high temporal consistency, especially given the spatial and temporal variability of spring migration. Fall migration exhibited greater temporal variability than spring migration. Our results support the hypothesis that higher fidelity to seasonal ranges is related to greater environmental and resource predictability. Interestingly, this fidelity was stronger at larger scales and at the population level. Almost the entire herd would seek out these areas with predictable resources, and then, individuals would vary their use, likely in response to annually varying conditions. During seasons with lower presumed spatial and/or temporal predictability of resources, population‐level fidelity was lower but individual fidelity was higher. The herd would be more spread out during the seasons of low‐resource predictability, leading to lower fidelity at the scale of their entire range, but individuals could be closer to locations they used the previous year, leading to greater individual fidelity, perhaps resulting from memory of a successful outcome the previous year. Our results also suggest that fidelity in 1 season is related to fidelity in the subsequent season. We hypothesize that some differences in patterns of range fidelity may be driven by seasonal differences in group size, degree of sociality, and/or density‐dependent factors. Climate change may affect resource predictability and, thus, the spatial fidelity and temporal consistency of use of animals to certain seasonal ranges.

## Graphical abstract

Fidelity to seasonal ranges was higher where there was greater environmental and resource predictability. Animals seek out these areas, but, once there, they would attenuate their selection likely due to interannual variation. Individuals may be able to locate and reuse patches of higher‐quality habitat within these larger regions of low‐resource predictability, increasing individual‐level fidelity.

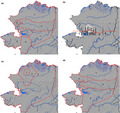

## 1 INTRODUCTION

Seasonal range fidelity, the tendency for animals to return to previously occupied areas (White & Garrott, 1990), is a common trait found across a wide array of taxa (Greenwood, 1980). Fidelity is thought to be dependent on habitat quality, density and behavior of conspecifics, degree of gregariousness, presence of predators, mating patterns, anthropogenic disturbance, and other factors (Faille et al., 2010; Gunn et al., 2012; Passadore et al., 2018; Peignier et al., 2019; Valkenburg et al., 1983; Wittmer et al., 2006). It has been suggested that high‐quality habitat is critical for the development of seasonal range fidelity (Passadore et al., 2018; Peignier et al., 2019; Schaefer & Mahoney, 2013). High fidelity to quality habitats could lead to increased survivorship and productivity, while low fidelity to them could reduce survivorship and/or productivity (Faille et al., 2010; Lafontaine et al., 2017). Similarly, high fidelity to low‐quality habitats could reduce survivorship and/or productivity (Lafontaine et al., 2017). Thus, fidelity has implications for maximizing fitness and optimal foraging theory (Giuggioli·& Bartumeus, 2012). The availability of high‐quality habitat must be predictable in order for high fidelity to develop (Passadore et al., 2018; Peignier et al., 2019), and the greatest fidelity should be to areas with the most predictable high‐quality habitat (Morrison et al., 2021). Habitat quality can be impacted by population density, and thus, population density can impact seasonal range fidelity (Taillon et al., 2012). Gregarious animals can take social cues from conspecifics which also can affect fidelity (Gunn et al., 2012; Peignier et al., 2019; Torney et al., 2018). Disturbance, either by actual or perceived predation pressure or human development, can cause animals to change their use of space or even abandon seasonal ranges (Faille et al., 2010; Passadore et al., 2018).

Animals’ space use, including seasonal range fidelity, varies by species, but also between individuals of the same species, and within an individual over time (Addicott et al., 1987). Degree of fidelity of an individual can be related to age, reproductive status, body condition, and/or social status (Passadore et al., 2018; Rettie & Messier, 2001; Schaefer et al., 2000; Wittmer et al., 2006). For social animals, studies of fidelity can be undertaken at the population level, identifying the ranges seasonally revisited by a larger group, and/or at the scale of the individual, by studying the interannual predictability of a single animal's movements.

**FIGURE 1:**
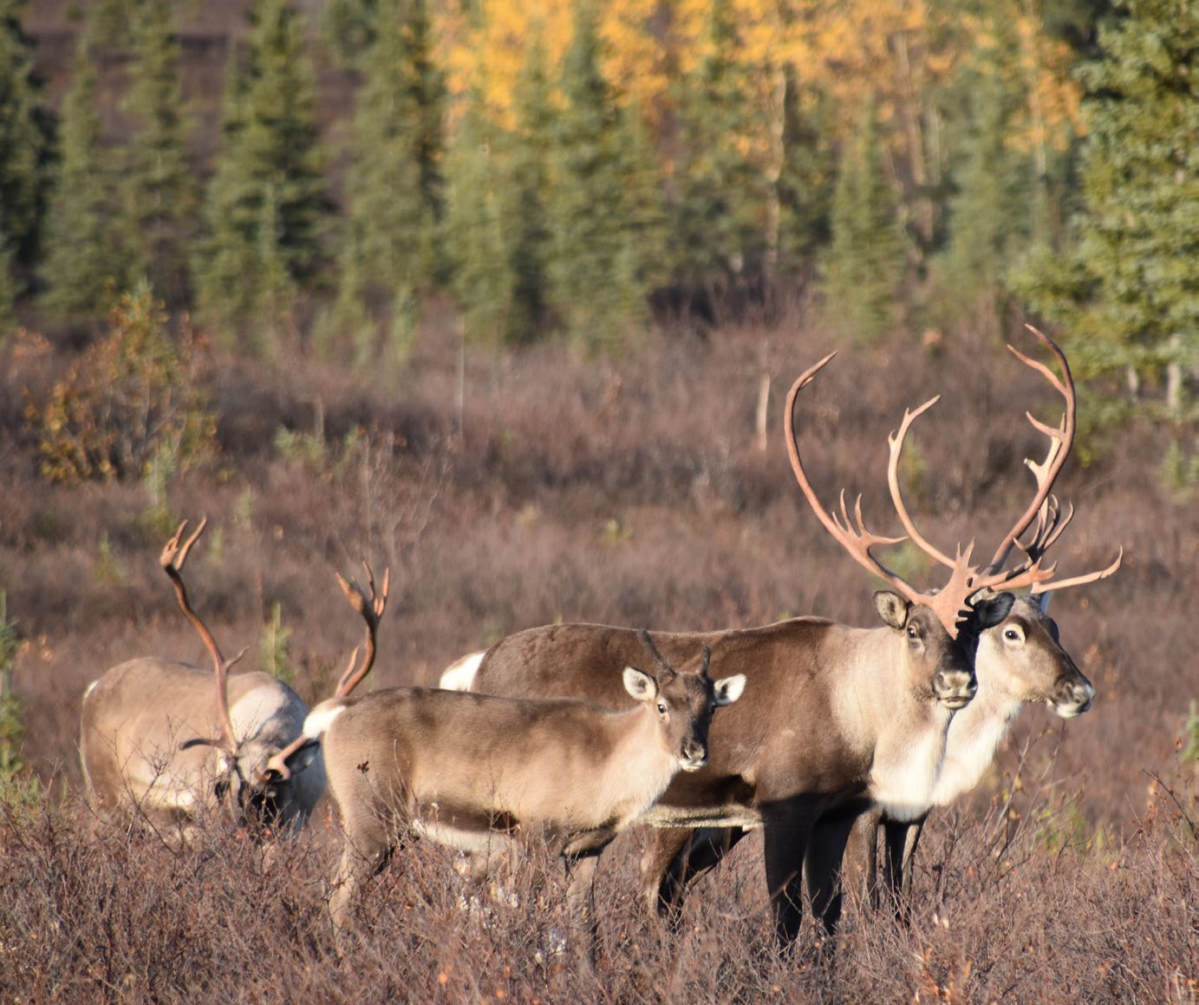
A group of caribou on their southward fall migration, Kobuk Valley National Park, northwest Alaska. Photo credit: Kyle Joly

Knowledge of seasonal range fidelity is important to understand an organism's ecology and to guide its conservation and management, specifically including the assessment and mitigation of anthropogenic impacts and delineating conservation areas and sound management practices (Giuggioli·& Bartumeus, 2012; Passadore et al., 2018). The annual return of caribou (*Rangifer tarandus*; Figure 1) to their calving grounds from their winter ranges is commonly used as an example of high fidelity to a seasonal range (Gunn & Miller, 1986) and is thought to be driven by access to areas of predictably high‐quality forage that is needed to restore body condition after winter and meet the demands of lactation (Cameron et al., 1993, 2020; Parker et al., 2009). Severe harassment by insects is known to have behavioral, physiological, and demographic impacts on caribou (Joly et al., 2020). The abiotic and less variable nature of what creates insect relief habitat (barren habitat, remnant snow patches, water, elevation; Joly et al., 2020) makes this resource, much like the calving grounds, more consistent in space and time.

Resources and environmental conditions tend to be more heterogeneous and unpredictable, but widespread, in the winter and late summer seasons than calving and insect relief seasons. In winter, patchily distributed and slow‐growing terricolous lichens comprise over 70% of the diet of barren‐ground caribou (Joly, 2011; Joly & Cameron, 2018; McMullin & Rapai, 2020). Lichen patches are vulnerable to wildfires and grazing when caribou densities are high, further increasing their patchiness across the landscape (Joly et al., 2010; Moser et al., 1979). Snow depth and density, which are important factors in winter caribou habitat selection, are also quite variable (Collins & Smith, 1991). In late summer, nutritional value of caribou forage tends to decline and, as selective feeders, caribou must cover large expanses to exploit high‐quality resources (Albon & Langvatn, 1992; Klein, 1990; White, 1983).

Fidelity to seasonal ranges shapes human use and interaction with caribou in many ways. Parturient females, and those with neonates, are displaced the most by anthropogenic disturbance, which is a primary reason conservation measures focus on calving grounds (Joly et al., 2006; Nellemann & Cameron, 1998; Taillon et al., 2012). However, the importance of seasonal range fidelity goes beyond assessing potential effects of anthropogenic impacts and development of conservation and mitigation measures for caribou calving grounds. While broadly defined as the seasonal return to particular locations or areas, fidelity also applies to the use of the same corridors for migration (Bond et al., 2017), which are of tremendous importance in terms of both human use of caribou and mitigating the impacts of development (Johnson et al., 2020; Plante et al., 2018; Wilson et al., 2016). Arctic Indigenous people, who depend on caribou for their material, cultural, and spiritual well‐being, would traditionally set up hunting camps in those places where the caribou were most reliably found, whether at the ends of their seasonal migration or at bottlenecks along migratory corridors and do so still today (Burch, 1972). Alternatively, hunters used an adaptive, nomadic strategy in those seasons where fidelity was lowest (Parlee et al., 2005). Thus, fidelity to migratory routes and winter ranges can directly and strongly influence accessibility of caribou and harvest levels. Hunters not only have to be at the right place, but they also need to be there at the right time for a hunt to be successful. Therefore, the temporal consistency of caribou movements and migrations was, and still remains, of paramount importance.

Effective monitoring and management of arctic caribou, given their remote and relatively inaccessible habitat, also depends on fidelity. Aerial population counts are typically conducted on the calving grounds (particularly in central Canada) or during peak insect harassment (particularly in Alaska and eastern Canada) when caribou are most tightly aggregated (Boulanger et al., 2011; Dau, 2015; Rivest et al., 1998). These population estimates rely on the observation that there is very high fidelity to these areas (i.e., that caribou that are counted belong to a specific herd and not to neighboring herds or that large segments of the population do not go uncounted). To this day, fidelity to calving grounds is the defining feature of arctic caribou herds (Skoog, 1968), which are the unit of management of barren‐ground caribou. Interchange of individuals among herds occurs at low levels and could also be affected by relative fidelity to seasonal ranges (Prichard et al., 2020). Herd overlap during the rutting period, which occurs in early stages of fall migration, may lead to substantial gene flow that increases genetic connectivity (Mager et al., 2013; Roffler et al., 2012).

Finally, a better understanding of current seasonal range fidelity may also aid researchers studying how species respond behaviorally to changes in forage availability, use spatial memory to relocate high‐quality habitat, niche separation of competing species, and the impacts of climatic change (Bartumeus et al., 2010; Giuggioli·& Bartumeus, 2012). Temperatures in the Arctic are warming faster than anywhere on the planet (Comiso & Hall, 2014), which has the potential to impact caribou in many ways (Joly & Klein, 2011; Mallory & Boyce, 2018). The temporal consistency of caribou migrations and calving events will likely be affected by climatic change but will depend on the degree of physiological and behavioral plasticity caribou have to adapt to these changes. Climate impacts that result in changes to habitat quality, predictability of resource availability, caribou density, and/or predator abundance could also affect seasonal range fidelity and temporal consistency of important life‐history events. Ultimately, fidelity can, in turn, impact all these things.

The goals of our study were to quantify the level of fidelity during 6 seasons for the Western Arctic Herd (WAH), a migratory caribou herd in northwest Alaska. We hypothesized that fidelity, across the range of the herd, would be highest during the calving and insect relief seasons and lowest during winter in concurrence with previous research (Faille et al., 2010; Gunn & Miller, 1986; Passadore et al., 2018; Peignier et al., 2019; Popp et al., 2011; Schaefer & Mahoney, 2013). This would also support the hypothesis that animals exhibit higher fidelity to areas with greater resource predictability (Morrison et al., 2021). “Resources,” as we understand it, encompasses not only forage but also other critical factors such as insect relief habitat, reduced predation, and other elements that can enhance fitness. Although we expected high range‐wide, population‐level fidelity to areas with presumed high‐resource predictability, we predicted that during late summer and winter, within large areas of presumed low‐resource predictability, individuals would show greater individual fidelity, as perhaps they were able to locate and return to known isolated patches of higher‐quality habitat within the lower‐quality matrix. We predicted that while fidelity in winter would be less pronounced than at calving, WAH caribou would show some fidelity to their winter ranges and, of these ranges, that fidelity to northern winter ranges would be low, perhaps due to lower abundance of forage lichens. We predicted that fidelity to migratory routes would be intermediate to that of calving and winter seasonal ranges. We predicted that the temporal consistency of calving would be high, despite high variability in the timing of spring migration. We also predicted that fall migration would exhibit even less temporal consistency than spring migration.

## 2 METHODS

### 2.1 Study area

**FIGURE 2:**
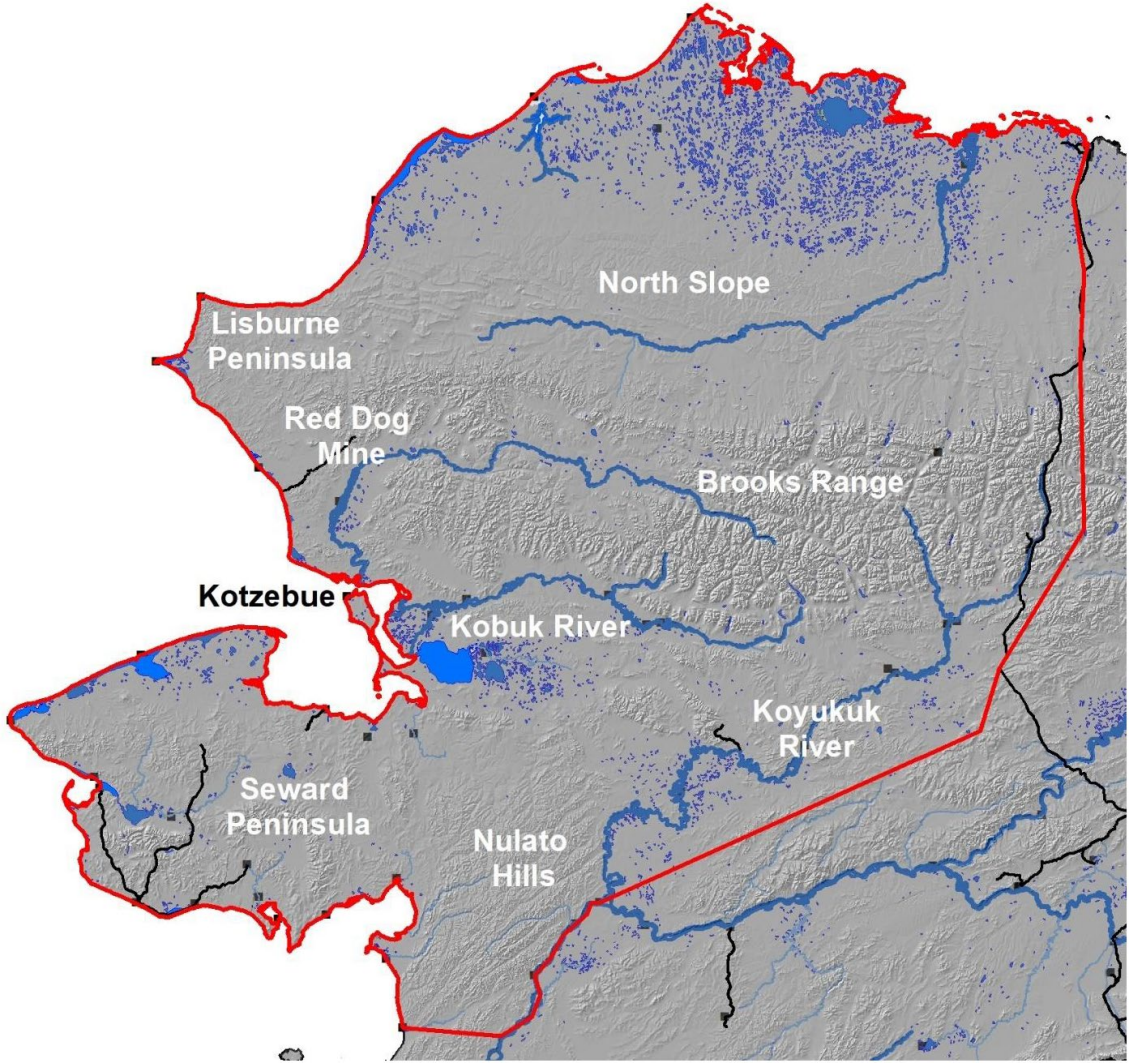
Study area, outlined in red, encompasses the range of the Western Arctic Herd, northwest Alaska, 2010–2019. Roads are depicted with black lines and villages with black squares

The WAH ranges over 360,000 km^2^ of northwestern Alaska (Figure 2). The WAH undergoes large population oscillations, recently ranging from a low of 75,000 individuals in 1976 to a peak of 490,000 in 2003 (Dau, 2015; Joly et al., 2011). During the study period, the population experienced general decline from ~348,000 (2010) to ~244,000 (2019; Dau, 2015; A. Hansen, unpublished data). The WAH's range is dominated by arctic and alpine tundra but also contains large tracts of boreal forest (Joly et al., 2007; Valkenburg et al., 1983). It is roughly bounded by the Chukchi Sea on the west, Beaufort Sea on the north, the Dalton Highway on the east, and the Koyukuk‐Yukon River system on the south. The topography is varied, from extensive lowland coastal tundra plains to rugged mountain peaks over 2,000 m in elevation. The mountains comprising the Brooks Range run roughly east to west across the range of the WAH. The mountains are rugged with extensive areas of rock and alpine tundra, with the eastern portion being more rugged with taller mountains. The western portion contains the Red Dog Mine, one of the world's largest lead and zinc mines. While there are small isolated villages and developments, this is the only major industrial development within the range of the herd. Valley bottoms often have narrow riparian corridors lined with willows (*Salix* spp.). The western Brooks Range is the source of several major rivers, including the Kobuk, Noatak, and Koyukuk. North of the Brooks Range, on Alaska's North Slope, the mountains give way to foothills, ridges, and, eventually, an expansive coastal plain, which is dominated by cottongrass (*Eriophorum* spp.) and underlined with permafrost. Parturient females primarily calve in the Utukok uplands, which is part of the North Slope (Cameron et al., 2020; Dau, 2015; Lent, 1966). Afterward, the entire herd gathers southwest of the calving grounds, at the very western edge of the Brooks Range, and can form huge (>100,000 individuals) aggregations during peak insect harassment (Joly et al., 2020). The Brooks Range and the North Slope are typically heavily utilized in summer. The Seward Peninsula and Nulato Hills were common wintering areas during the study period. The Seward Peninsula is more diverse, with isolated mountain ranges reaching just over 1,200 m, extensive arctic tundra, wetlands, and large lakes. The Nulato Hills are more uniform, dominated by worn but somewhat rugged hills and smaller tributaries. Much of the remainder of the area is at the periphery of the herd's range and is dominated by interior boreal forest of the Koyukuk River drainage.

### 2.2 Caribou location data

We affixed GPS collars on adult (primarily >2 years old) caribou as they swam across the Kobuk River in Kobuk Valley National Park during their southward, fall migration from 2009 to 2018 (Dau, 2015; Joly et al., 2012). All captures were in accordance with established and approved animal handling protocols (State of Alaska IACUC 0040‐2017‐40). Caribou data were not used until the individual was considered randomly mixed with the herd (i.e., calving season the year following its fall capture; Joly et al., 2012). Collars collected data spanning 2009–2019 on at least 8‐hr intervals. The initial data pool was over 500,000 relocations from 240 individual female caribou, and the temporal coverage for an individual ranged from 2 to 7 years.

### 2.3 Seasonal ranges

**FIGURE 3:**
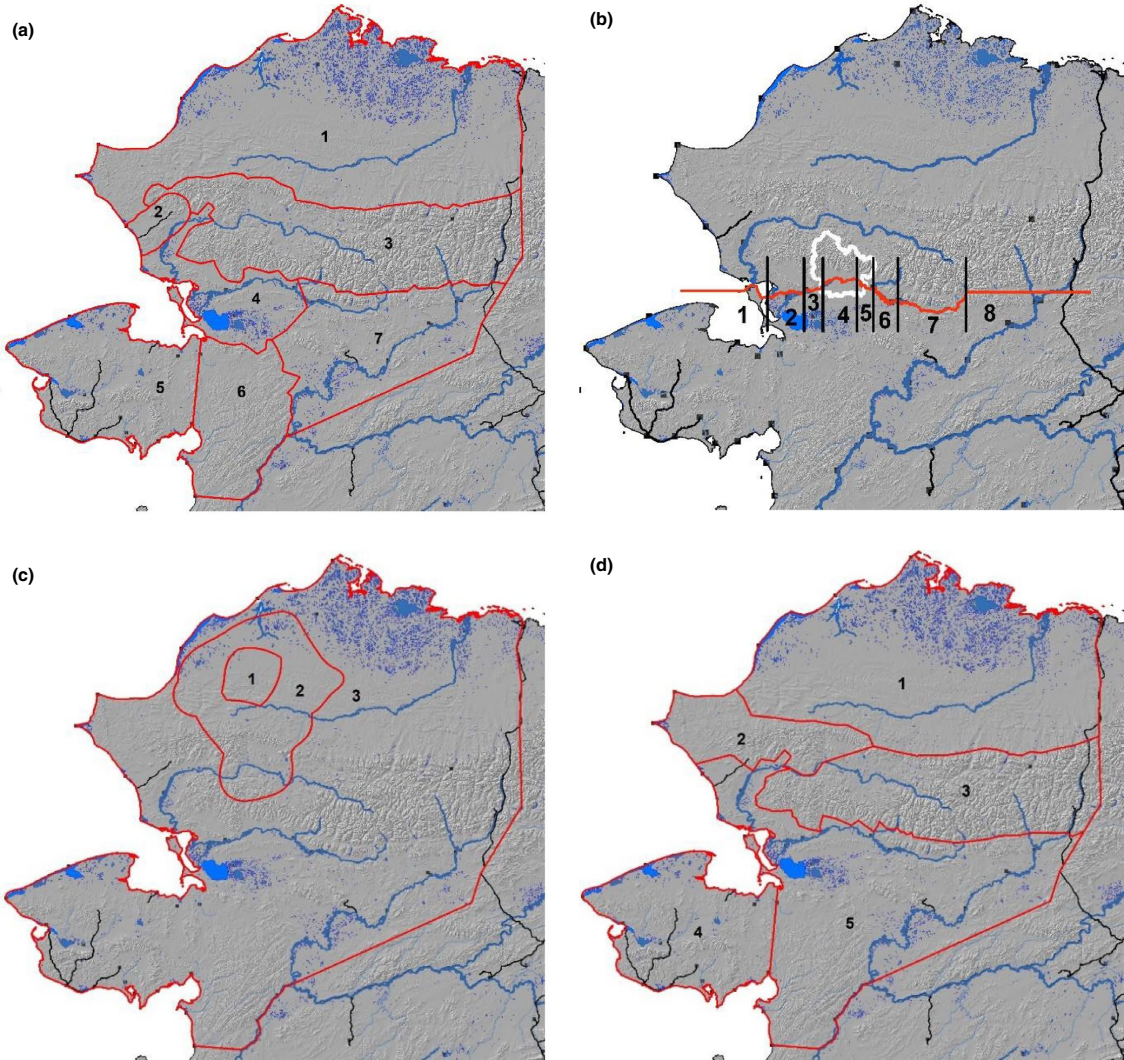
Six seasonal ranges of the Western Arctic Herd, northwest Alaska, 2010–2019. (a) winter, (b) spring and fall migratory corridors with the boundary of Kobuk Valley National Park in white, (c) calving grounds, and (d) insect relief and summer. Red lines delineate extent of subareas for the various seasonal ranges, except for migratory corridors, used to explore patterns of fidelity by female caribou. The red line for the migratory seasons delineates the Kobuk River, and westward and eastward extensions of it, used to capture migratory crossings. Black lines separate these different corridors. Numbers correspond to regions. Winter: (1) North Slope, (2) Red Dog, (3) Brooks Range, (4) Kotzebue Lowlands, (5) Seward Peninsula, (6) Nulato Hills, and (7) Koyukuk. Migratory Corridors: (1) Ocean, (2) Ocean to village of Kiana, (3) Kiana to western boundary of Kobuk Valley National Park, (4) Kobuk Valley to Hunt River, (5) Hunt River to Ambler River, (6) Ambler River to Kobuk village, (7) Kobuk to Walker Lake, and (8) east of Walker Lake. Calving grounds: (1) Core Calving, (2) Extent of Calving, and (3) Remainder. Insect relief and Summer: (1) North Slope, (2) Western Brooks Range/Lisburne, (3) Central Brooks Range, (4) Seward Peninsula, and (5) Remainder

We divided the year into 6 biologically meaningful seasons: winter, spring migration, calving, insect relief (early summer), late summer, and fall migration (Figure 3). For each season, the entire range of the herd was divided into regions dependent on our collective understanding of the spatial ecology of the WAH (Figure 3). We included a region surrounding the Red Dog Mine and its port road in the winter season because liver and kidney caribou samples from this area during winter have elevated concentrations of lead and cadmium (Garry et al., 2018). For spring and fall migration, the Kobuk River (a major river bisecting the herd's migratory range) was delineated into river length segments rather than areas (Figure 3). One location per animal per year was selected for each season. For winter (5 January), peak insect harassment (5 July), and late summer (5 August), locations were chosen for a specific date that was representative of the given season. We chose the location closest to the start of the selected day, and 98.6% locations were within 24 hr of the desired date and time. Spring migration, calving, and fall migration locations were chosen based on events. For spring and fall migration, locations were based on when they first crossed the Kobuk River. An event‐based method was chosen for migration because the timing of migration is highly variable and, in recent years, the percentage of collared caribou not migrating to the wintering grounds they have used for the last couple of decades has increased (Joly & Cameron, 2019). We also chose an event‐based method for calving as nonparturient females often do not reach the calving grounds (Dau, 2015; Joly, 2011).

### 2.4 Tests of fidelity by season

**FIGURE 4:**
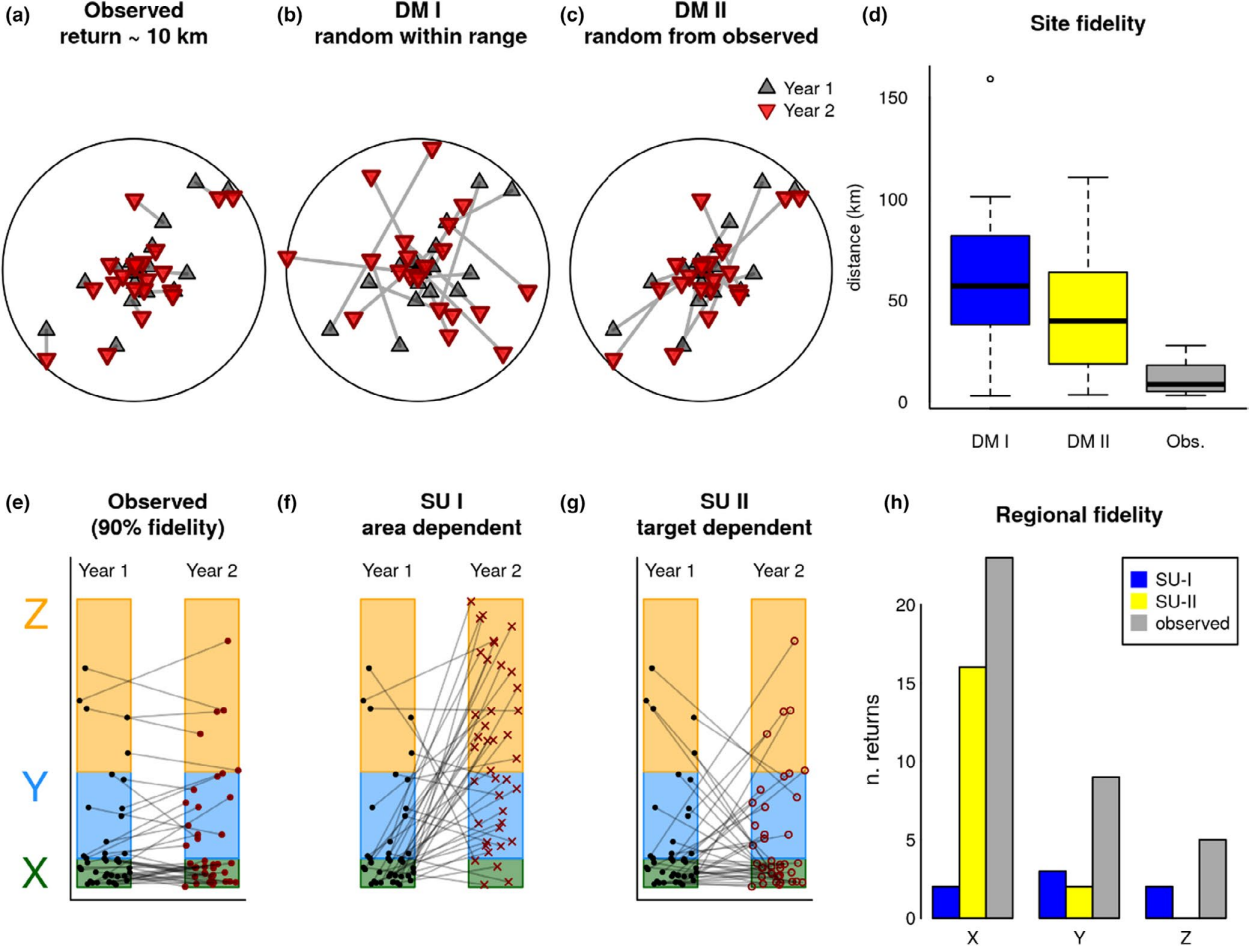
Illustration of distance metric (DM) fidelity analyses at the site scale and space use (SU) fidelity at the regional scale. Both scales compared observed caribou relocations with 2 different null hypotheses. For site fidelity (a), the first hypothesis (*range‐conditioned distance metric*, DM I) allowed random relocations to fall anywhere within the range of the Western Arctic Herd (b). Under the second hypothesis (*location‐randomization distance metric*, DM II), random relocations were constrained to where actual caribou were observed, but these relocations were randomized (c). For regional fidelity (e), the first (*range‐conditioned space use*, SU I) hypothesis (f) assumed that the number of random locations was proportional to the relative area of the region (e.g., X, Y, and Z). The second hypothesis (*location‐randomization space use*, SU II) assumed that the number of returns to the same region would be proportional to the number of observations (targets) from that region (g) from the previous year (Year 1). We used 90% fidelity as an example here for the observed data. Site fidelity (d) would occur if the distance between successive relocations was smaller than the observed distances. Regional fidelity (h) would occur if the number of returns exceeded the number observed. For this example, fidelity was not shown at either scale. The spatial scale of our analyses increases from DM I to DM II SU I to SU II

Given the importance of scale in considering caribou fidelity (Schaefer et al., 2000), we performed 2 broad analyses each with a hierarchy of scales, as detailed in the subsections below. In summary, (1) we compared the actual distance between locations used by individuals across subsequent years (Figure 4a) to 2 distance metric (DM) null sets. For the first null set, randomized locations were generated that could fall anywhere within the herd's range (*range‐conditioned*, DM I; Figure 4b), while the second was a randomization of the observed caribou locations (*location randomization*, DM II; Figure 4c). Next, (2) we compared space use (SU) of actual caribou locations across subsequent years (Figure 4d) to assess caribou fidelity to discrete regions across the herd's range that were identified a priori. Randomized locations for the first null set could fall anywhere within the herd's range but were in proportion to the relative size of the region to the herd's range (*range‐conditioned*, SU I; Figure 4e); and the second null set randomized the observed locations (*location randomization*, similar to DM II), thereby reflecting the actual distribution of large‐scale space use (SU II; Figure 4f). These different approaches allowed us to assess fidelity at very fine scales to the largest scale of the entire herd's range. Further details of the fidelity tests are below.

### 2.5 Distance metrics (DM) of site fidelity

For a metric of site fidelity, we examined Euclidean distances between an individual's location across subsequent years (henceforth, interannual individual distances (IIDs)) for each of the 6 seasons and compared them against 2 randomized null sets in a manner similar to the methods suggested by Schaefer et al. (2000). The first null hypothesis was a set of random points from within the herd's entire range (Dau, 2015; Joly et al., 2011), which is analogous to Schaefer et al.'s (2000) “population‐range” scale. For each season, we generated 100 sets of random points for each caribou, with each set having the same number of relocations as the corresponding individual. We referred to this set as the *range‐conditioned distance metric* (DM I). We then computed all the paired IIDs between the actual caribou locations and the corresponding simulated locations.

The second null hypothesis was a more conservative randomization of the actual caribou locations in a given season and compared the distribution of consecutive distances across years with the complete set of possible distances between locations across years. In other words, we compared the actual interannual distances across consecutive years for each individual against distances between the location of each animal in a given year with every observed location of all other caribou in the subsequent year (in effect, randomizing the individual). We referred to this metric, analogous to Schaefer et al.'s (2000) “seasonal‐population‐range” scale, as the *location‐randomization distance metric* (DM II). We illustrated DM I and DM II in Figure 4. Statistical differences between observed successive caribou relocations and DM I and DM II relocations for each season were assessed using *t* tests of the mean distance of the observed and corresponding null sets.

### 2.6 Space use (SU) regional fidelity metric

We estimated the probabilities of an animal in a given subregion in a given season returning to that subregion or moving to another subregion the subsequent year using a transition probability matrix. In the jargon of Markov processes, both returns to the same region (i.e., fidelity) and switches to different regions (i.e., lack of fidelity) are referred to as “transitions.” Because we were interested in space use fidelity (i.e., the probability of return), our analysis was focused on transitions across years back to the same region (i.e., an observation that an animal summering in region X in year *t* returns to that subregion in year *t + *1). However, we completed the entire transition probability matrix (including transitions to different regions). After obtaining these transitions across all years of data for each of the 6 seasons, we compared those transitions against 2 different null hypotheses. The first null hypothesis assumed that the probability of transitioning was proportional only to the area of the region relative to the entire study area, such that:

(1) $$\mathrm{Pr}\left(X_{t}=j\vee X_{t‐1}=i\right)=\frac{A_{j}}{\sum_{i}^{k}A_{i}}$$

where *X_t_* and *X_t_*_−1_ were the observed regions in years *t*−1 and *t* respectively, *A_j_* was the area of region *j*, and the sum was over all the regions. For example, if there were three regions X, Y, and Z with areas corresponding to 10%, 30%, and 60% of the total area, those percentages corresponded to the null set of transition probabilities. The unconditional probability of a given transition from region *i* to region *j* was given by:

(2) $$P_{\mathrm{ij}}=\mathrm{Pr}\left(x_{t}=j,x_{t‐1}=i\right)=\left(\frac{A_{j}}{\sum A_{j}}\right)\left(\frac{N_{i,t‐1}}{\sum N_{i,t‐1}}\right)$$

where *N_i_*_,_ *_t_*_−1_ represented the number of individuals in region *i* in the previous year, and the sums, as above, were taken over all of the regions. The set of all transitions *i* to *j* was then compared with the multinomial distribution from the complete set of *k* ^2^ unique transitions in Equation 2. As an example, consider an individual starting in region X with a null probability of 10%, 30%, and 60% of showing up in regions X, Y, and Z, respectively, the following year based on the areas of these subregions. If 60 of 100 of the complete set of observed transitions begin in X, the unconditional (complete) null absolute probability of animals transitioning from X to X, Y, and Z would be 6%, 18%, and 36%. We referred to this as the *range‐conditioned space use test* (SU I, Figure 3).

A more conservative test of regional fidelity used as a null set of probabilities the actual observed end points (*targets*) of interannual transitions:

(3) $$P_{\mathrm{ij}}=\left(\frac{N_{j,t}}{\sum N_{j,t}}\right)\left(\frac{N_{i,t‐1}}{\sum N_{i,t‐1}}\right)$$

This test was equivalent to randomizing all of the target locations (*X_j,t_*) relative to all the source locations (*X_i,t_*_−1_) and assessing whether certain transitions occur more frequently than expected, again using the resulting multinomial distribution as the null. We referred to this test as the *location‐randomization* *space use test* (SU II). In both cases, we were most interested in transitions to the same site, which we defined as space use fidelity at the regional scale. In Figure 4, we illustrated both null hypotheses against a simulated set of observations with relatively high (90%) regional fidelity and compared the observed number of returns against the nulls.

For both SU I and SU II, statistical significance was assessed by comparing the number of observed transitions across a pair of regions against a binomial distribution with the corresponding null probability. We reported observed and expected numbers of transitions, differences in the respective probabilities, and corresponding *p*‐values.

### 2.7 Temporal consistency of migration and calving dates

We calculated the date of event for migrations and calving. Dates used for migration were the day an individual caribou first crossed the Kobuk River heading north in spring or south in fall. Calving events were detected based on GPS movement data using methodology developed specifically for this herd by Cameron et al. (2018). We determined repeatability (*R*), the proportion of total variation that is reproducible among repeated measurements of the same group, of these dates (Nakagawa & Shielzeth, 2010). If *R* = 0, individuals are individually random, with no mean differences among individuals. If *R* = 1, then individuals behave identically from year to year. We calculated *R* using generalized linear mixed‐effects models via the “rptR” package in *R* (Stoffel et al., 2017). We also quantified the number of caribou that did not migrate south across the Kobuk River, as it appears the number that do not has been increasing (Joly & Cameron, 2019).

## 3 RESULTS

### 3.1 Site fidelity using the range‐conditioned distance metric (DM I)

IIDs (mean ± *SE*) were shortest during calving (57.0 ± 16.1 km) and greatest during the winter (226.8 ± 10.2 km; Table 1). As with calving, spring and fall migration IIDs were under 100 km in all years but note that the migration distances were constrained linearly to the river itself. Distances between successive caribou locations were significantly shorter than the distances between random locations for all seasons (Table 1).

**TABLE 1:** Average interannual individual distances (IIDs; km ±*SE*) between actual individual Western Arctic Herd caribou (observed) and random relocations across the herd's range (*range‐conditioned distance metric*, DM I) in successive years for six different seasons, northwest Alaska, 2010–2019

Season	Observed	Random (DM I)	*T*	*p*
Winter	226.8 ± 10.2	348.2 ± 1.0	−11.82	<.001
Spring migration	74.9 ± 10.7	152.3 ± 1.1	−7.20	<.001
Calving	57.0 ± 16.1	346.9 ± 1.6	−17.96	<.001
Insect relief	85.4 ± 11.4	349.2 ± 1.1	−23.02	<.001
Late summer	136.4 ± 7.5	209.6 ± 0.7	−9.69	<.001
Fall migration	77.8 ± 9.6	152.8 ± 0.9	−7.74	<.001

### 3.2 Site fidelity using the location‐randomization distance metric (DM II)

**FIGURE 5:**
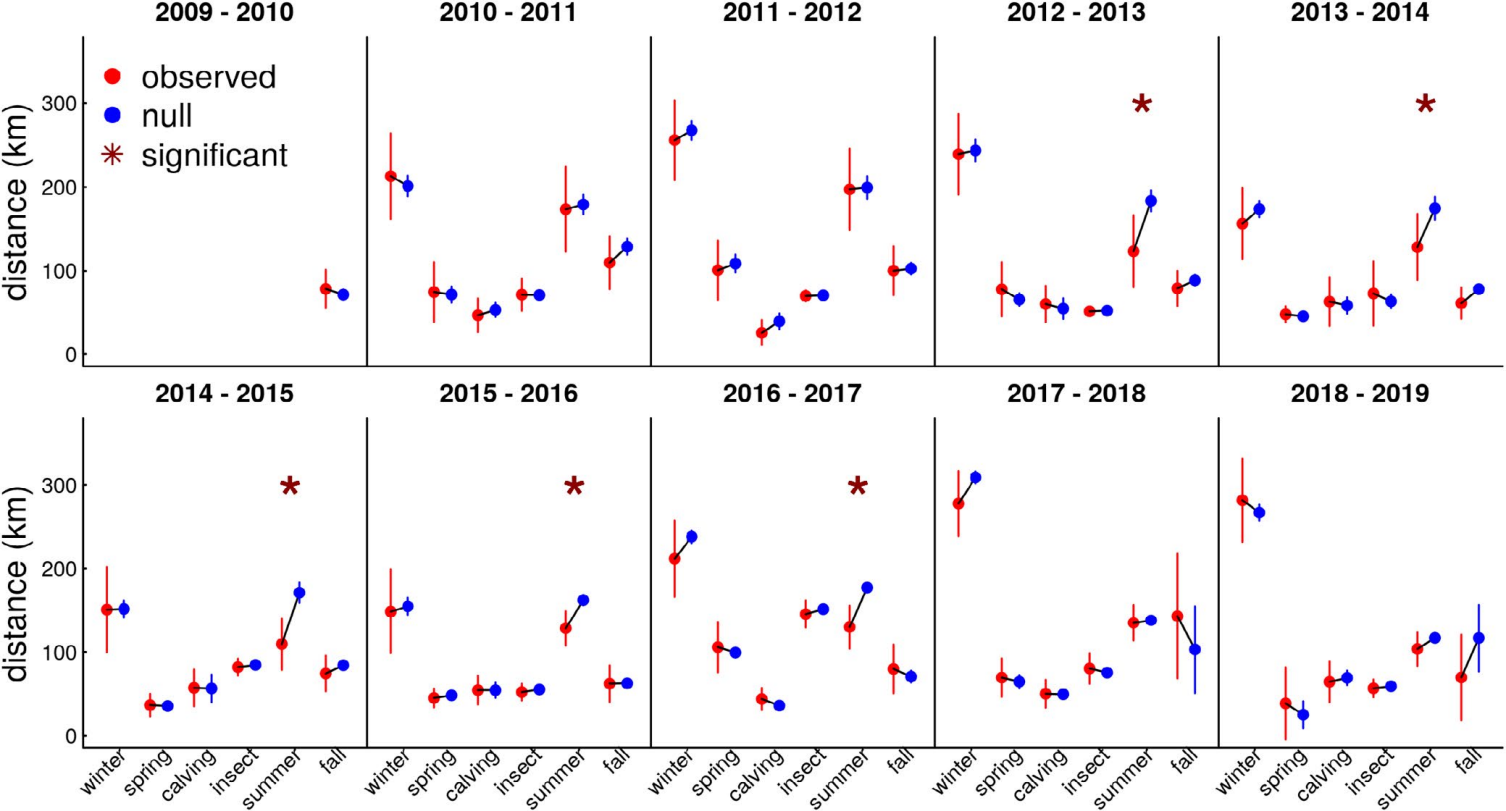
Interannual individual distances (IIDs) between observed successive relocations of Western Arctic Herd caribou (red) compared with distances between randomized caribou relocations (blue; *location‐randomization distance metric* DM II) for 6 different seasons in northwest Alaska, 2009–2019. Asterisks denote a statistical difference in the distances

IIDs of observed caribou were not significantly different from those of randomized caribou locations (Figure 5) except for the late summer season. In all summers, observed caribou IIDs were smaller than DM II, and in 4 of the 7 years, the difference was significant (*p* <.05). There was also considerable interannual variation for all seasons (Figure 5). Distances were greatest during winter and summer, while the other seasons were relatively similar.

### 3.3 Regional fidelity using the range‐conditioned space use (SU I) test

At the largest scale and relative to total availability across the range, caribou showed significant fidelity to at least 1 region or migratory corridor for each of the 6 seasons (Table 2). In winter, fidelity was greatest in the Seward Peninsula region, but also statistically significant in the Brooks Range and Nulato Hills (Table 2). The number of returns was significantly lower than expected for the North Slope. In spring, 3 migration corridors (Ocean to Kiana, Kiana to Kobuk Valley National Park, and Hunt River to Ambler) were reused significantly more than expected. Caribou showed very high fidelity to their core calving grounds. In both the insect relief and late summer seasons, caribou showed strong fidelity to the western Brooks Range/Lisburne region, but not other regions (Table 2). In fall, caribou showed the greatest fidelity to the corridor between the Hunt River and Ambler, but high fidelity was also demonstrated to the corridor between Kiana and Kobuk Valley National Park.

**TABLE 2:** Relative size (or length), use (relative number of caribou relocations), and fidelity (reuse) of various regions (number in parenthesis correspond to Figure 3) within the range of the Western Arctic Herd for 6 seasons, northwest Alaska, 2010–2019

Season	Region	Size/length (% of total)	Caribou locations (% of total)	Observed reuse	Null	Sign	*p*
Winter	North Slope (1)	37.2	7.4	6	10.79	−	.046
	Red Dog (2)	1.5	1.8	0	0.03		.970
	Brooks Range (3)	19.7	13.7	19	6.7	+	<.001
	Kotzebue (4)	6.5	5.3	1	0.84		.583
	Seward Peninsula (5)	12.5	51.4	124	30.25	+	<.001
	Nulato Hills (6)	8.8	14.4	13	6.07	+	.007
	Koyukuk (7)	13.8	6.1	4	2.76		.295
Spring migration	Ocean (1)	16.6	7.9	1	2.82		.200
	Ocean to Kiana (2)	11.4	32.9	28	7.64	+	<.001
	Kiana to Kobuk Valley N.P. (3)	5.2	18.1	11	2.24	+	<.001
	Kobuk Valley N.P. to Hunt River (4)	10.0	20.2	6	4.10		.223
	Hunt River to Ambler (5)	5.0	15.0	6	1.65	+	.005
	Ambler to Kobuk (6)	7.5	2.9	0	0.45		.626
	Kobuk to Walker Lake (7)	20.5	1.7	0	0.62		.502
	Walker Lake and East (8)	23.8	1.4	0	0.24		.762
Calving	Core Calving (1)	1.8	65.0	50	1.49	+	<.001
	Calving Extent (2)	11.4	34.2	7	3.76		.075
	Remainder (3)	86.9	0.8	0	0.87		.132
Insect relief	North Slope (1)	33.8	0.7	0	0.97		.267
	W. Brooks Range/Lisburne (2)	6.6	98.9	307	29.36	+	<.001
	Central Brooks Range (3)	17.0	0.2	0	0.00		1.000
	Seward Peninsula (4)	12.5	0.2	0	0.18		.821
	Remainder (5)	30.1	0.0	0	0.00		1.000
Late summer	North Slope (1)	33.8	55.6	109	109.26		.512
	W. Brooks Range/Lisburne (2)	6.6	5.6	79	17.09	+	<.001
	Central Brooks Range (3)	17.0	38.7	0	0.00		1.000
	Seward Peninsula (4)	12.5	0.2	0	0.24		.764
	Remainder (5)	30.1	0.0	0	0.00		1.000
Fall migration	Ocean (1)	16.6	7.7	1	2.83		.189
	Ocean to Kiana (2)	11.4	8.5	2	2.24		.606
	Kiana to Kobuk Valley N.P. (3)	5.2	7.5	4	1.23	+	.031
	Kobuk Valley N.P. to Hunt River (4)	10.0	4.6	3	1.57		.202
	Hunt River to Ambler (5)	5.0	58.6	59	10.96	+	<.001
	Ambler to Kobuk (6)	7.5	9.3	5	2.85		.151
	Kobuk to Walker Lake (7)	20.5	3.4	1	2.42		.257
	Walker Lake and East (8)	23.8	0.4	0	0.00		1.000

Note

Caribou fidelity results presented here were based on the *range‐conditioned space use* (SU I) test. “Observed Reuse” is the actual number of caribou that returned to that region the following year, and ‘Null” is the expected number of returns based on the relative size of the region. A positive sign (+) under “Sign” indicates fidelity (more returns than expected) and a negative sign (−) a lack of fidelity (less returns than expected). “*p*” indicates strength of statistical significance.

### 3.4 Regional fidelity using the location‐randomization space use (SU II) test

**FIGURE 6:**
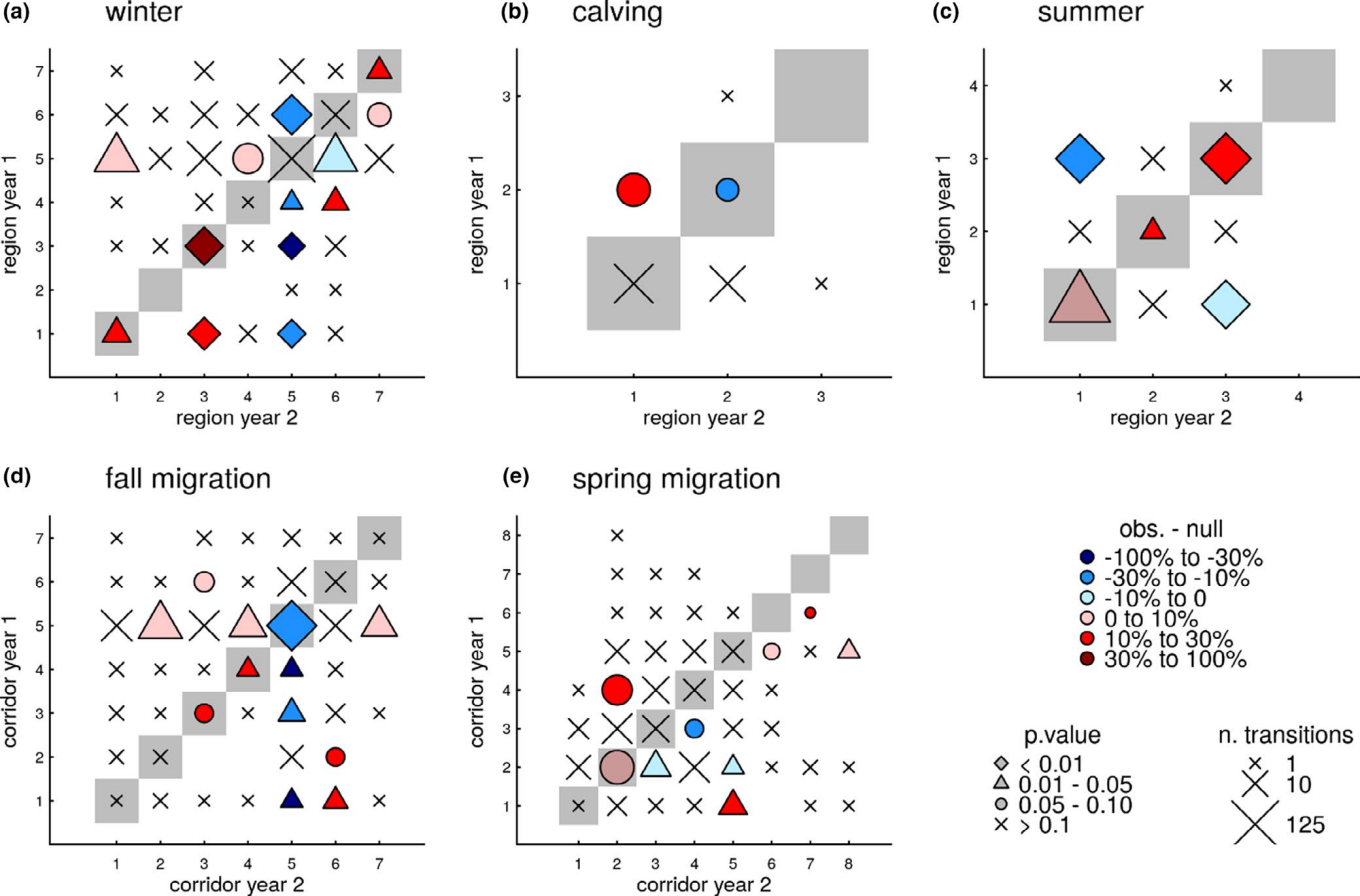
Plots of Western Arctic Herd caribou fidelity, using the *location‐randomization space use* (SU II) test, to different seasonal ranges, northwest Alaska, 2010–2019. The *x*‐axis is the location of the caribou in Year 1, and the *y*‐axis is the region in Year 2. The 1:1 diagonal (gray‐shaded squares) represents fidelity, a return to the same region, based on the “expected null.” The numbers of the regions/corridors correspond to the number designations found in Figure 2. The shape of the symbol depicts its statistical significance level, the color is the difference between the observed and null, and the size is the sample size. The matrix could not be developed for the insect relief season because almost all locations were within the Western Brooks/Lisburne region (i.e., there was virtually no variation and complete fidelity). Regions that had no transitions are also not reported

Fidelity to winter ranges was low, except for the few animals that were significantly more likely to return to the low‐use Brooks Range, North Slope, and Koyukuk regions (Figure 6a). North Slope wintering caribou that did not return the following year tended to overwinter in the adjacent Brooks Range the following winter significantly more than expected. Interestingly, caribou from various previous wintering areas did not use the Seward Peninsula significantly more than expected the following winter. Seward Peninsula wintering caribou ended up on the North Slope the following winter significantly more than expected and in the adjacent Nulato Hills significantly less than expected.

Fidelity to regions during calving was not detected at this scale, and instead, we found a tendency for caribou to calve within the core calving grounds if they calved out of it the previous year (Figure 6b). During the insect relief season, 98.9% of the locations were within the western Brooks Range/Lisburne region. This shows a tremendous amount of fidelity to the region; in fact, too few relocations occurred in other regions to even run the randomization test for this season. During late summer, caribou showed significant fidelity to the Central Brooks Range, North Slope, and Western Brooks Range regions (Figure 6c). Caribou summering in the Central Brooks Range were significantly less likely to summer in the North Slope region the next year and vice versa. There was low fidelity to most fall migratory corridors, and in fact, we found a significant probability for caribou to not reuse the Hunt River to Ambler corridor in the subsequent fall migration (Figure 6d). One exception was significant fidelity to the corridor between the western boundary of Kobuk Valley National Park and the Hunt River (Figure 6d). Not all caribou migrated across the Kobuk River every year. Nonmigratory caribou, in a given year, had a 46% chance of being nonmigratory the following year, which was significantly (*p* < .01) higher than the null expectation (21%). In contrast, caribou that did migrate were somewhat, though significantly (*p* < .01), less likely to migrate in the following year compared with the null expectation (73.5% vs. 78.4%). In other words, migratory animals were that much more likely to not migrate in the subsequent year compared with the null (26.5% vs. 21.6%). There was no observed, significant (*p* < .05) fidelity to spring migration corridors, and use of the various corridors was well distributed (Figure 6e).

### 3.5 Temporal consistency of migration and calving dates

We detected crossings of the Kobuk River by 187 individual caribou in spring, with a mean date (and *SD*) of May 9 (±9.0 days), ranging from 20 April to 7 June. For the 124 individual caribou that crossed repeatedly, the average difference in spring crossing dates between successive years was 13.8 ± 11.9 days. We detected calving events for 148 individual caribou from 27 May to 12 June, with a mean calving date of 3 June (±3.4 days). Individual parturient females (*n* = 91) displayed high consistency to calving date: The average difference between successive calving events for individual caribou was 3.8 ± 2.7 days. Interestingly, 90.1% of females had their calving events take place ≤7 days apart, on average, while 68.1% were ≤4 days apart, and 15.4% ≤1 day apart. The average date of crossing the Kobuk River in fall for 187 individual caribou was 28 September (±12.7 days), ranging from 3 September to 11 November. For 145 individual caribou that crossed repeatedly (i.e., in multiple sequential years), the average difference in fall crossings dates between successive years was 24.8 ± 18.8 days. For spring migration, *R* = 0.067 ± 0.052 and was not significantly (*p* = .073) different from 0. For calving, *R* = 0.351 ± 0.076, which was significantly (*p* < .001) greater than 0. For fall migration, *R* = 0.000 +/ 0.027 and was not significantly (*p* = 1) different from 0. The average duration between fall migration and the subsequent spring migration (i.e., the length of time between when they crossed the Kobuk River in the fall and then again in the spring) for 183 individual caribou that stayed south of the Kobuk River was 226 ± 16.1 days. For the 124 caribou that repeatedly overwintered south of the Kobuk and returned north, the average difference in the duration they stayed on their wintering grounds between successive years was 27.8 ± 21.6 days.

## 4 DISCUSSION

### 4.1 Regional and site fidelity

How species utilize the landscape is paramount for understanding their ecology and conservation. Patterns of fidelity, or repeated use of places within a population's range, can have ecological, demographic, and management implications (Giuggioli·& Bartumeus, 2012; Lafontaine et al., 2017; Passadore et al., 2018). Caribou in large herds are among the most vagile terrestrial species on the planet and can have vast ranges (Joly, et al., 2011, 2019). They also inhabit arctic and subarctic environments that are extremely seasonal, making them a robust candidate for a detailed analysis of seasonal range fidelity. Though simple, the metrics we developed succinctly characterize the interannual process of fidelity at large and small scales. The distance metric (DM) requires minimal a priori assumptions and can address the fine‐scale question of return to specific sites. The space use (SU) metric has the advantage of identifying specifically *which* regions are associated with the highest levels of fidelity.

Our results add to existing research documenting high fidelity of caribou to their core calving grounds and postcalving insect relief habitats (Cameron et al., 1986; Gunn & Miller, 1986; Nagy et al., 2011; Schaefer et al., 2000; Skoog, 1968). High fidelity to calving grounds is the distinguishing and defining feature of caribou herds (Skoog, 1968), and WAH caribou have shown fidelity to their core calving grounds for at least 100 years (Cameron et al., 2020; Lent, 1966). WAH caribou showed highly significant fidelity to their core calving grounds at the regional scale, and this fidelity has been linked to areas with predictable high‐quality vegetation (Cameron et al., 2020).

Virtually, all caribou aggregated in the Western Brooks Range/Lisburne region and during the insect relief season. The region consistently provides cool winds coming off the ocean (which has just recently thawed at this time of year), as well as sparsely vegetated hills, lingering snow patches, and aufeis that provides some relief from harassing insects (Joly et al., 2020). Thus, our results support the hypothesis that predictability of critical resources enhances fidelity at the regional scale. Given that neonatal caribou have lower mobility than adults, greater usage of western areas of predicted high‐quality calving habitat may be related to their proximity to insect relief habitats that the herd utilizes just after calving (Cameron et al., 2020).

Observed IIDs during both the calving (57 km) and insect relief (85 km) seasons were significantly less than expected by random (DM I). For calving, this distance was very similar to that reported for the Porcupine Caribou Herd in northeast Alaska (67 km; Fancy & Whitten, 1991) but less than half the distance reported for the George River Herd in Quebec (123 km; Schaefer et al., 2000). While <60 km is a relatively small distance for a herd that ranges over 360,000 km^2^ (Joly et al., 2007), in terms of fidelity to a specific calving site, it is not particularly close. Boreal caribou in Canada are known to calve within 4–12 km of their previous calving locations (Popp et al., 2011; Schaefer et al., 2000). Moreover, using our SU II approach, WAH caribou did not show fidelity to the calving regions we delineated, but rather a slight trend for caribou calving in the greater extent of the calving grounds to move into the core the next year. This agrees with Cameron et al. (2020), who showed the core calving area for WAH tends to shift from year to year depending on annual variability in habitat quality. Likewise, the insect relief IIDs averaged about 85 km. So, while caribou fidelity to regions with high‐resource predictability, such as their calving grounds and insect relief areas, is more pronounced at larger spatial scales (implying they regularly seek out specific areas during these seasons), once they reach these regions, they attenuate their selection to maximize forage quality or insect relief at smaller spatial scales, likely in response to fine‐scale, interannual environmental and resource variability and stochastic events. In other words, the population, in general, shows strong fidelity to these regions with high‐resource predictability, but then individuals utilize the region depending on annual conditions, which lowers fidelity at that scale. In other ungulates, such as moose (*Alces alces*), high levels of individual calving site fidelity appears to be related to reproductive output (Tremblay et al., 2007; Welch et al., 2000). Given the lack of apparent spatial fidelity in calving sites, it is unlikely that calving site fidelity at a finer scale than the slowly shifting core calving ground plays a significant role in reproductive output, though this question requires further directed research.

Not surprisingly, IIDs were greatest during winter (227 km) and late summer (136 km; Table 1), seasons that are believed to have lower resource predictability (Faille et al., 2010; Peignier et al., 2019; Schaefer et al., 2000). However, we identified some fidelity to certain regions and some fidelity to sites within regions in those seasons as well. Caribou showed significant fidelity to the Seward Peninsula, Nulato Hills, and the Brooks Range, but returned to the North Slope less than expected during winter. The first 2 of these regions were heavily utilized in the earlier years of the study and contain high‐quality winter range characterized by lichen‐rich habitats (Joly, 2011; Joly & Cameron, 2018, 2019). Moving to different winter areas each year may allow caribou to avoid heavily grazed areas. Use of the Brooks Range and North Slope, which are thought to be lower‐quality winter range (Joly, 2011; Joly & Cameron, 2018, 2019), increased toward the end of our study period. Interestingly, caribou wintering in 3 of the low‐quality ranges (North Slope and Brooks Range, which is attributed to low lichen biomass, and Koyukuk, which is attributed to deep snow) showed significant fidelity to these areas using the SU II approach. Given how few individuals were responsible for this relationship (Figure 6a), we posit that these caribou were able to locate adequate, isolated patches of quality habitat and benefitted from a lack of intraspecific competition (density‐dependent resource limitations). This may be an example where enhanced knowledge of an individual's range, acquired from fidelity, allows for use of small higher‐quality habitat patches within a larger poorer‐quality habitat matrix. Alternatively, these areas could be acting as populations sinks. Our study coincided with a population decline, which may have also affected range utilization.

Late summer relocations were limited to the tundra‐dominated North Slope, Central Brooks Range, and Western Brooks Range/Lisburne regions. Caribou showed significant fidelity to the Western Brooks Range/Lisburne region using both the regional (SU I and SU II) approaches. Our results support previous research, which revealed the importance of vast tracts of tundra for summering caribou (Klein, 1970; Russell et al., 1993). After a 6‐month winter, when access to protein is limited, the demands of lactation and intense insect harassment result in female caribou that can be in poor body condition. In late summer, female caribou seek to gain as much mass as possible to reach an adequate body condition to be able to become pregnant and have enough stores to last the long winter (Cameron et al., 1993; Parker et al., 2009). Notably, in late summer and, to a lesser degree, winter, individual caribou were found closer to where they were the year before than to other caribou (DM II). Caribou are herd animals and highly social. Female group sizes tend to be larger during calving and insect relief seasons than in winter and summer, when they tend to be the smallest. We posit that the differences in patterns of fidelity between summer/winter and calving/insect relief may reflect herd‐ versus individual‐level responses, sociality, and/or density‐dependent factors, as well as resource and environmental predictability. In other words, while individual caribou tend to show some level of fidelity across all these seasons, the repeated use by most members of the herd of their calving grounds and insect relief areas accentuates fidelity during these seasons, while individualism is stronger during winter and late summer. The distribution of key resources may also play an important role. Calving (high‐quality forage) and insect relief (cool, windy areas) resources are relatively small, concentrated areas of high‐quality habitat. During late summer and winter, in contrast, the overall quality of forage may be lower and distributed more patchily. Attraction to known high‐quality preferred patches may explain the higher level of individual site fidelity, even as the population itself spreads over a larger area. This is consistent with theoretical studies that have found that spatial memory is most beneficial for foragers in landscapes where resources are most patchily distributed (Bracis et al., 2015).

Migration corridors were well distributed along the length of the Kobuk River, but fall migration was more variable than spring. In both spring and fall migration, corridors that we found caribou displayed fidelity to matched up with those identified by Baltensperger and Joly (2019). There was no discernable fidelity to any spring migration corridor except at the largest scale (SU I), which showed fidelity to 3 corridors that were further west than those identified for fall. In fall, fidelity to various corridors was method‐dependent. Using the SU I approach, caribou showed not only the greatest fidelity to the corridor between the Hunt and Ambler Rivers but also the significant fidelity to the corridor between Kiana and western boundary of Kobuk Valley National Park. Using the SU II approach, we detected significant fidelity to the fall migration corridor located between the western boundary of Kobuk Valley National Park and the Hunt River. Surprisingly, the corridor between the Hunt and Ambler Rivers had significantly fewer returns the following year than expected. This stretch of river is considered to reliably experience the highest number of caribou crossing events (Joly et al., 2012). A possible explanation of this result is that caribou are disturbed along this stretch and therefore avoid it the following year. We doubt this hypothesis because this stretch was significantly less likely to be used by caribou that crossed 3 other corridors the previous year. Further research on this inconsistency is warranted.

We surmise that fidelity in 1 season may be related to fidelity in the next season. For example, where caribou wintered may preposition them relative to where they are likely to cross the Kobuk River during spring migration and the relative lack of fidelity to winter ranges regulates the likelihood of fidelity to spring migratory routes. Similarly, we posit that the high variability in the use (or lack of use, i.e., nonmigration) of fall migration corridors factors into the relatively low fidelity in winter, particularly if fall caribou movements are indeed as exploratory as they are migratory (Fullman et al., 2017).

Migration is a behavioral adaptation to maximize fitness given fluctuations of resource availability, both in space and in time (Dingle & Drake, 2007). Migration patterns of a population can be a continuum between resident and migrant behavior at the individual level that gives rise to partially migratory populations (Ball et al., 2001; Cagnacci et al., 2011). For migratory populations that exhibit partial migration, where only a proportion of individuals exhibit migratory movements (Cagnacci et al., 2011; Chapman et al., 2011), individuals may either be obligate migrants (migrate every year) or facultative migrants (vary migration strategies each year based on conditions; Dingle & Drake, 2007). Based on our simple definition of migration (i.e., individuals cross the Kobuk River), our results reveal partial migration for the WAH, with some individuals exhibiting facultative migration.

The classic definition of migration is based on consistent, annual movement patterns between discrete seasonal ranges (e.g., winter and calving grounds or wet and dry seasonal ranges). While WAH caribou consistently use their core calving grounds, they show low fidelity to winter ranges and thus do not fit neatly into the classic definition of migratory. Recently, Teitelbaum and Mueller (2019) provided a framework to classify annual movements that fell outside of the stereotypical migration patterns (consistent annual patterns between discrete areas) as differing degrees of nomadism. Studying annual trends in range use (fidelity) of populations provides a quantitative means to apply the classifications proposed by Teitelbaum and Mueller (2019), and the framework we developed (DM I, DM II, SU I, SU II) here should allow researchers to pursue this topic across a wide array of taxa. Given that WAH caribou exhibit some level of fidelity for all seasons, are not territorial, and do not fit into the range of nomadic movements described by Teitelbaum and Mueller, further exploration of space use (range residency, migration, nomadism) theory is warranted.

### 4.2 Temporal consistency

The timing of spring migration in caribou is somewhat plastic and likely related to snow conditions and nutritional status of individuals (Gurarie et al., 2019; Laforge et al., 2021). We found that WAH caribou, on average, crossed the Kobuk River on 9 May, with a range of 20 April to 7 June, on their northbound spring migration. Late migrants are likely nonparturient females that tend to lag behind parturient females during migration (Dau, 2015; Joly, 2011). Individual caribou averaged about 2 weeks’ difference in the dates they crossed the Kobuk River in successive springs. Caribou that migrate later in the year can make up ground by traveling faster than caribou migrating early (Gurarie et al., 2019).

Temporal consistency of calving was very high. The average difference between successive calving events was <4 days, and >90% of females had their calving events take place ≤7 days apart. Given the variability in the environment, habitat, and timing of spring migration (Gurarie et al., 2019), this individual consistency to calving date is remarkable. In comparison, at the population level, calving spanned 12–20 days (Cameron et al., 2018). One possibility for the high synchrony is that caribou calving is linked, either as a consequence of or in anticipation of the short flush of high‐quality forage that emerges at this time of year (Cameron et al., 2020). Additionally, it could potentially facilitate “swamping” or numerically overwhelming neonatal predators (Williams et al., 1993; Young & McCabe, 1998). However, predation on WAH neonates on the calving grounds is relatively low (A. Hansen, unpublished data).

The average fall crossing date of the Kobuk River was 28 September. Very late crossing caribou tended to cross the frozen waters of Kotzebue Sound in the far west of our study area. The range in crossing date was much greater in fall (a span of 69 days) than spring (48 days). Similarly, for those caribou that crossed the Kobuk River in the subsequent year, the average difference in fall crossing dates between successive years (25 days) was also much greater than in spring (14 days). We attribute this greater variability in timing to the high variability in the timing of substantive snow accumulation in the fall, in contrast to the pressing urge to reach the calving grounds in the spring (Gurarie et al., 2019; Le Corre et al., 2017). Caribou that migrated across the Kobuk River were significantly less likely to migrate again the following year, and those caribou that failed to migrate across it the first year were significantly more likely to not migrate again the following year. This result also supports the observation that fewer caribou overall are migrating across the Kobuk River in fall (Joly & Cameron, 2019). WAH caribou stayed an average of 226 days (over 7 months) south of the Kobuk River. As calving, photo‐censuses, and the majority of harvest occurs on and north of the Kobuk River, considerable management attention is focused there; however, our results show the relative importance of ranges south of the Kobuk River to the ecology of the WAH during our study period. Declining numbers of caribou crossing the Kobuk River in fall may alter this relationship.

Future research that assesses insect relief season fidelity when caribou are most tightly aggregated, rather than a set date like we used here, may provide valuable insights. Caribou are incredibly mobile and can cover large distances in relatively short order (Joly et al., 2019), so caribou may have fidelity to certain locations, such as insect relief, but the timing of its use may vary, affecting its estimated fidelity depending on methodology. Both event‐based and set‐date methodologies have advantages and disadvantages that vary spatiotemporally and likely as well due to differences in study areas if our methods were transferred elsewhere. Furthermore, we expect that in seasons with low movements, such as mid‐winter for caribou, these differences would be muted while being accentuated in seasons with greater movement rates (insect harassment, spring migration, and fall migration; Joly et al., 2020).

### 4.3 Management implications

Understanding seasonal range fidelity can have direct management implications. For example, given the strong and remarkably long pattern of fidelity of WAH caribou to their calving grounds, this area should factor into considerations regarding conservation efforts. Alternatively, a breakdown in calving ground fidelity can have important management and societal impacts (Adamczewski et al., 2015). While our analyses focused on females, male caribou, which typically migrate later than parturient females, consistently join the females and their neonates during the insect relief season. As a result, fidelity to the most consistently used insect relief habitats tends to involve the entire herd. These areas currently lie largely outside of conservation units. The consistency and predictability with which calving and insect relief habitats are used, as well as the relatively small areas they constitute, allow for targeted conservation efforts. In contrast, the low winter range fidelity exhibited by caribou means that vast tracts of winter range are used to sustain large herds of these ungulates. Retaining connectivity may be more appropriate for maintaining the functionality of extensive winter ranges.

Another management implication related to our work is that many populations of caribou are known to have elevated levels of heavy metal contaminants, such as lead and cadmium—particularly in specific organs such as the liver and kidneys (Garry et al., 2018). This is an important concern because many subsistence‐based communities consume high levels of caribou and these contaminants can bioaccumulate (Kuiters, 1996). Patterns of fidelity can affect the amount of exposure to potential contaminant sources, and a better understanding of these patterns could help mitigate impacts that may occur via human consumption of caribou. For example, if caribou showed high fidelity to an area around a potential contaminant source, mitigation measures could be proposed to reduce exposure. Alternatively, if fidelity was low, it may suggest that the caribou are accumulating the contaminants elsewhere. In our study, we found low fidelity to winter ranges, specifically the Red Dog Mine region. Liver and kidney tissues of caribou sampled from the Red Dog Mine region have elevated concentration of lead and cadmium (Garry et al., 2018). A concern was that caribou may be acquiring them by feeding near the mine site winter after winter. However, we did not have a single caribou return to this region in the subsequent or any other year during winter. Thus, we do not believe repeated use of habitat near the mine during winter is why WAH caribou have elevated levels of lead and cadmium. Alternative possible explanations for elevated contaminant levels include that they are acquiring them during other seasons or in different regions altogether. This would be consistent with the widespread findings of elevated levels of some bioaccumulating heavy metals in *Rangifer* across the Arctic (see Garry et al., 2018). Given the relatively low fidelity to winter ranges by WAH caribou, elevated concentrations of contaminants in their tissues may be an issue for users across the herd's range, which includes about 40 rural villages and hunters coming from outside northwest Alaska.

Our findings, along with previous studies, have shown that fidelity can be complex, varying at both different spatial and temporal scales. Similarly, there are myriad ways of analyzing fidelity. We recommend future work attempt to define a lexicon for fidelity analyses to improve standardization and enhance comparability of research efforts.

### 4.4 Limitations of our research

We did not empirically assess resource predictability; thus, while our results support the hypothesis that fidelity is linked to resource predictability (Morrison et al., 2021; Passadore et al., 2018; Peignier et al., 2019), we could not directly test this relationship. For the winter, insect relief, and late summer seasons, we used locations from a single day. This methodology is prone to spatial outliers, as individuals may display greater fidelity if a larger temporal window was utilized (sensu Morrison et al., 2021). However, spatial outliers may be related to phenological differences among years, whose magnitude is likely related to resource predictability. Further, the larger the temporal window that is utilized, the more locations there will be, inflating the chance of a type II error. Our methodology is conservative, and applying other methods to our data would likely result in the identification of even greater fidelity. Fidelity to seasonal ranges will vary among species, populations, and even within a population over time; thus, while our methodology can be applied widely, extrapolating our results to other places or times need to be done with caution.

## 5 CONCLUSIONS

Our results lend support to the theory that greater fidelity to seasonal ranges is linked to greater predictability in resource availability (Passadore et al., 2018; Peignier et al., 2019). Seasons with lower fidelity (winter and late summer) are characterized by both high‐resource heterogeneity and environmental variability, as well as widespread distribution. In contrast, seasons with high fidelity (calving and insect relief) may have greater resource and/or environmental consistency and limited distribution (Cameron et al., 2020; Joly, 2011). The climate and environment of the Arctic are rapidly changing, which could impact patterns of environmental variability and alter patterns of fidelity to seasonal ranges, and, thus, affect people that utilize the herd (Comiso & Hall, 2014; Swanson, 2017; Tape et al., 2006). We show that a greater understanding of movement ecology, including fidelity, can inform management decisions. This knowledge can also be used to develop species conservation plans and mitigation measures for development scenarios.

## CONFLICT OF INTEREST

The authors have no conflicts of interest to declare.

## AUTHOR CONTRIBUTION

**Kyle Joly:** Conceptualization (lead); Data curation (equal); Formal analysis (equal); Funding acquisition (lead); Investigation (equal); Methodology (equal); Project administration (lead); Resources (equal); Supervision (lead); Validation (equal); Visualization (equal); Writing‐original draft (lead); Writing‐review & editing (equal). **Eliezer** **Gurarie:** Conceptualization (equal); Formal analysis (equal); Investigation (equal); Methodology (equal); Project administration (equal); Validation (equal); Visualization (equal); Writing‐original draft (equal); Writing‐review & editing (equal). **D. Alexander Hansen:** Conceptualization (supporting); Funding acquisition (equal); Investigation (supporting); Methodology (supporting); Resources (equal); Writing‐review & editing (equal). **Matthew D. Cameron:** Conceptualization (supporting); Data curation (equal); Formal analysis (supporting); Investigation (supporting); Methodology (supporting); Resources (supporting); Writing‐review & editing (equal).

## Data Availability

Data generated during this study are included in this published article. Underlying GPS data used to generate these results are permanently archived within the National Park Service's Integrated Resource Management Applications (IRMA; https://irma.nps.gov/DataStore/Reference/Profile/2260262) and may be made available from the corresponding author on a case‐by‐case basis, in conjunction with the researcher(s) managing those data, and in accordance with respective legal constraints.
